# The neuroanatomy of *Barentsia discreta* (Entoprocta, Coloniales) reveals significant differences between bryozoan and entoproct nervous systems

**DOI:** 10.1186/s12983-019-0307-z

**Published:** 2019-03-28

**Authors:** Anastasia O. Borisanova, Vladimir V. Malakhov, Elena N. Temereva

**Affiliations:** 1 Biological Faculty, Dept. Invertebrate Zoology, Moscow State University, Leninskie Gory, 1-12, 119991 Moscow, Russia; 2 Far Eastern Federal University, 690600 Vladivostok, Russia

**Keywords:** Bryozoa, Kamptozoa, α-Tubulin, Confocal laser-scanning microscopy, Evolution, Ganglion, Nervous system, Phylogeny, Serotonin, Ultrastructure

## Abstract

**Background:**

Entoprocta affinities within Lophotrochozoa remain unclear. In different studies, entoprocts are considered to be related to different groups, including Cycliophora, Bryozoa, Annelida, and Mollusca. The use of modern methods to study the neuroanatomy of Entoprocta should provide new information that may be useful for phylogenetic analysis.

**Results:**

The anatomy of the nervous system in the colonial *Barentsia discreta* was studied using immunocytochemistry and transmission electron microscopy. The ganglion gives rise to several main nerves: paired lateral, aboral, and arcuate nerves, and three pairs of tentacular cords that branch out into tentacular nerves. The serotonergic nervous system includes paired esophageal perikarya and two large peripheral perikarya, each with a complex net of neurites. Each tentacle is innervated by one abfrontal and two laterofrontal neurite bundles. Sensory cells occur regularly along the abfrontal side of each tentacle. Star-like nerve cells are scattered in the epidermis of the calyx. The stalk is innervated by paired stalk nerves.

**Conclusions:**

The neuroanatomy of the colonial *Barentsia discreta* is generally similar to that of solitary entoprocts but differs in the anatomy and ultrastructure of the ganglion, the number of neurite bundles in the calyx, and the distribution of serotonin in the nerve elements. A comparison of the organization of the nervous system in the Entoprocta and Bryozoa reveals many differences in tentacle innervations, which may indicate that these groups may not be closely related. Our results can not support with any certainty the homology of nervous system elements in adult entoprocts and adult “basal mollusks”.

## Background

The relationships between Entoprocta and other taxa remain unclear, despite numerous morphological and molecular studies. The current hypothesis that Entoprocta and Cycliophora are closely related is generally accepted based on both morphological and molecular data [1–5]. In a number of molecular studies, Entoprocta and Cycliophora are considered to be a sister group to Bryozoa (=Ectoprocta) [5–7]. Some researchers have suggested, however, that a close phylogenetic relationship between Entoprocta and Bryozoa may be an artifact due to compositional bias [8, 9]. Morphological data also do not provide an unambiguous answer to the question about Entoprocta affinities within Lophotrochozoa. Based on morphology, some authors unite them with Bryozoa [10–12], while other authors consider Entoprocta to be unrelated to bryozoans [13, 14] but perhaps closely related to annelids [15] or mollusks [16–18]. To some extent, such contradictions result from the insufficient knowledge of the details of Entoprocta internal anatomy, which has been inadequately investigated by modern methods. For example, the nervous system of Entoprocta has been mainly investigated with light microscopy. A paired ganglion, which gives rise to several pairs of peripheral nerves passing to internal organs, was described in investigated species of entoprocts [13, 19–22]. A recent investigation using immunocytochemical staining has shown that the neuroanatomy of entoprocts is much more complex than previously indicated [23]. The authors of the latter report studied two solitary species and described paired oral, aboral, and lateral nerves; three pairs of tentacular nerves; as well as the nerves of the calyx, stomach, rectum, esophageal nerve ring, and atrial nerve ring. To date, this has been the only detailed investigation of the neuroanatomy of Entoprocta. Ultrastructural data on the nervous system of entoprocts is extremely fragmentary [15, 21, 24].

Here, we investigated the nervous system of colonial species *Barentsia discreta* using transmission electron microscopy and immunocytochemistry with subsequent confocal laser scanning microscopy. Our objective was to obtain new data on the organization of the entoproct nervous system that can be used to determine the phylogenetic position of this group.

## Results

### General morphology

**Fig. 1:**
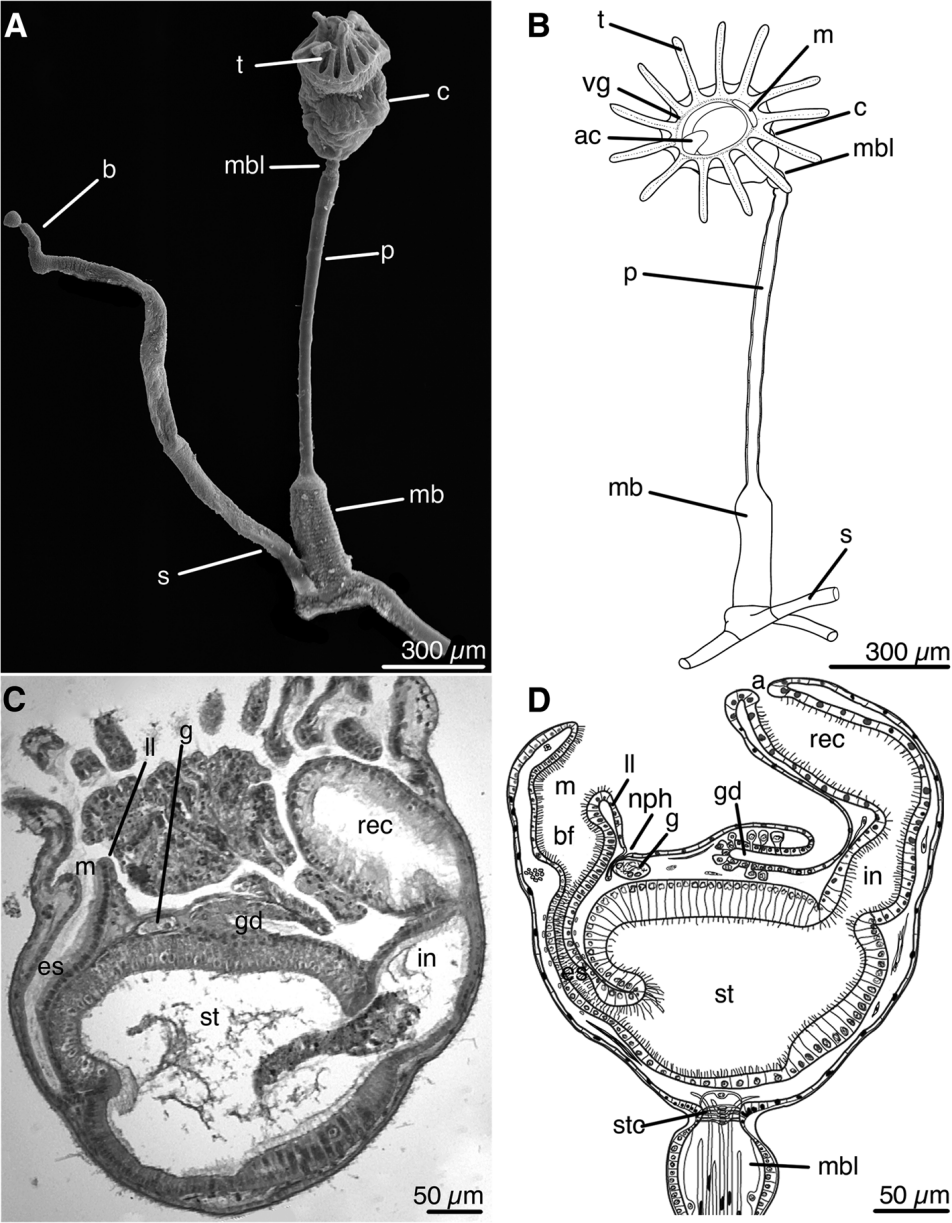
General morphology of *Barentsia discreta*. **a** Part of a colony with a zooid and young bud (SEM), (**b**) Scheme of the zooid, (**c**) Histological sagittal section of the calyx, (**d**) Scheme of a sagittal section of the calyx. Abbreviations: a, anal opening; ac, anal cone; b, bud; bf, buccal funnel; c, calyx; es, esophagus; g, ganglion; gd, gonoduct; in, intestine; ll, lower lip, m, mouth; mb, muscular base; mbl, muscular bulbous; nph, nephropore; p, peduncle; rec, rectum; s, stolon; st, stomach; stc, star-cell complex; t, tentacle; vg, vestibular groove

*Barentsia discreta* is a colonial species with a branching, creeping stolon (Fig. 1a, b). Each zooid consists of a calyx and a stalk, separated by a cuticular septum. A star-cell complex is connected with the septum. The calyx usually bears 14–16 tentacles, but some individuals have up to 20 tentacles. The stalk is divided into a muscular bulbous, which is located directly under the calyx, a thin rigid peduncle, and a muscular base. All internal organs are in the calyx (Fig. 1c, d). The space surrounded by the tentacles is called the atrial cavity. The bottom of the atrial cavity is a ventral wall of the body. At the base of the tentacles, there is a ciliary vestibular groove that leads to a slit-shaped mouth on the frontal side of the calyx (Fig. 1b). The mouth leads to the esophagus, starting with an enlarged buccal funnel. The esophagus opens into a bulky stomach, followed by an intestine and a rectum. The rectum is located in the muscular anal cone, which protrudes into the atrial cavity (Fig. 1c, d). A ganglion is located next to the back wall of the esophagus, behind the mouth and above the stomach (Fig. 1c, d). It is transversely elongated relative to the antero-posterior axis. Paired protonephridia are located on the sides of the calyx. Nephridial channels meet one another and merge into the common excretory duct, which opens with a single excretory opening located in the sagittal plane between the ganglion and the lower lip of the mouth (Fig. 1d).

### Ganglion

**Fig. 2:**
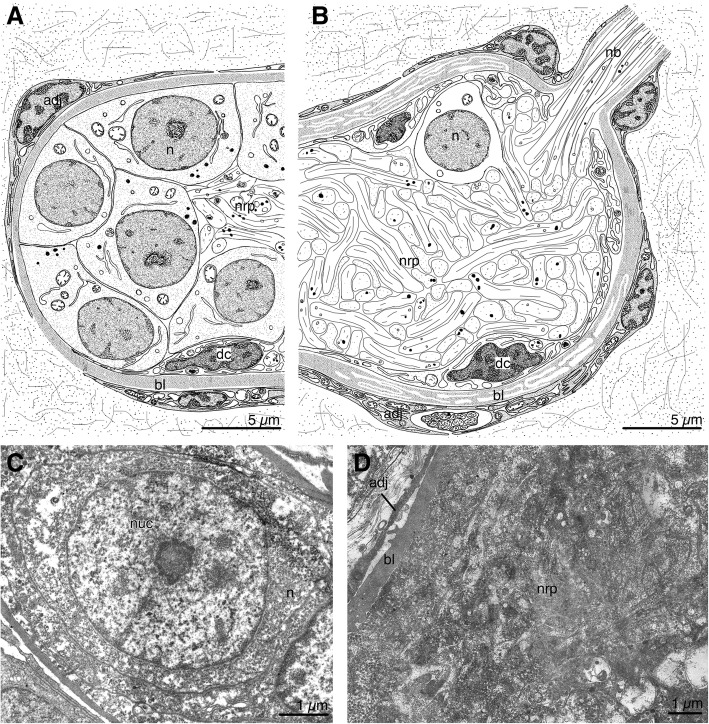
Ultrastructure of the ganglion of the calyx in *Barentsia discreta*. **a** Diagram of the longitudinal section through the ganglion on the level of the ganglion periphery, (**b**) Diagram of the longitudinal section through the ganglion on the level of the ganglion central zone, (**c**) A perikaryon of the ganglion, (**d**) The neuropil of the ganglion. Abbreviations: adj, adjoining cell of a body cavity; bl, basal lamina; dc, dark cell of ganglion; n, nerve cell; nb, neurite bundle; nuc, nucleus; nrp, neuropil

The ganglion of *B. discreta* is oval-shaped, with a slight depression in the center. The ganglion is 60–70 μm long with a transverse diameter of about 20 μm. Perikarya are located on the periphery of the ganglion and form two hemispheres on its sides, separated by a space that does not contain perikarya. The central portion of the ganglion is filled with numerous neurites (Fig. 2a, b). The nerve cells in the ganglion total about 40–60. Each nerve cell is about 10–12 μm. There are two types of cells in the ganglion. The first type are nerve cells that have transparent cytoplasm and large, rounded nuclei with finely dispersed chromatin and several nucleoli, located in the centre of the cell body (Fig. 2c); the cytoplasm contains an endoplasmic reticulum, mitochondria, and synaptic vesicles with electron-lucent content. The second type are dark cells that have round or oval nuclei, with a predominance of heterochromatin (Fig. 2a, b); the cytoplasm is dense, with a well-developed reticulum and vacuoles with granular contents. The dark cells have long processes that stretch along the basal lamina or penetrate into the neuropil. In the neuropil, many nerve processes are intertwined with each other (Fig. 2b, d). The ganglion is surrounded by a thick layer of extracellular matrix (basal lamina) with a complex structure and by adjoining cells of the body cavity (see [25]).

The ganglion does not exhibit serotonin-like immunoreactivity or tubulin-like immunoreactivity.

### Innervation of the calyx

**Fig. 3:**
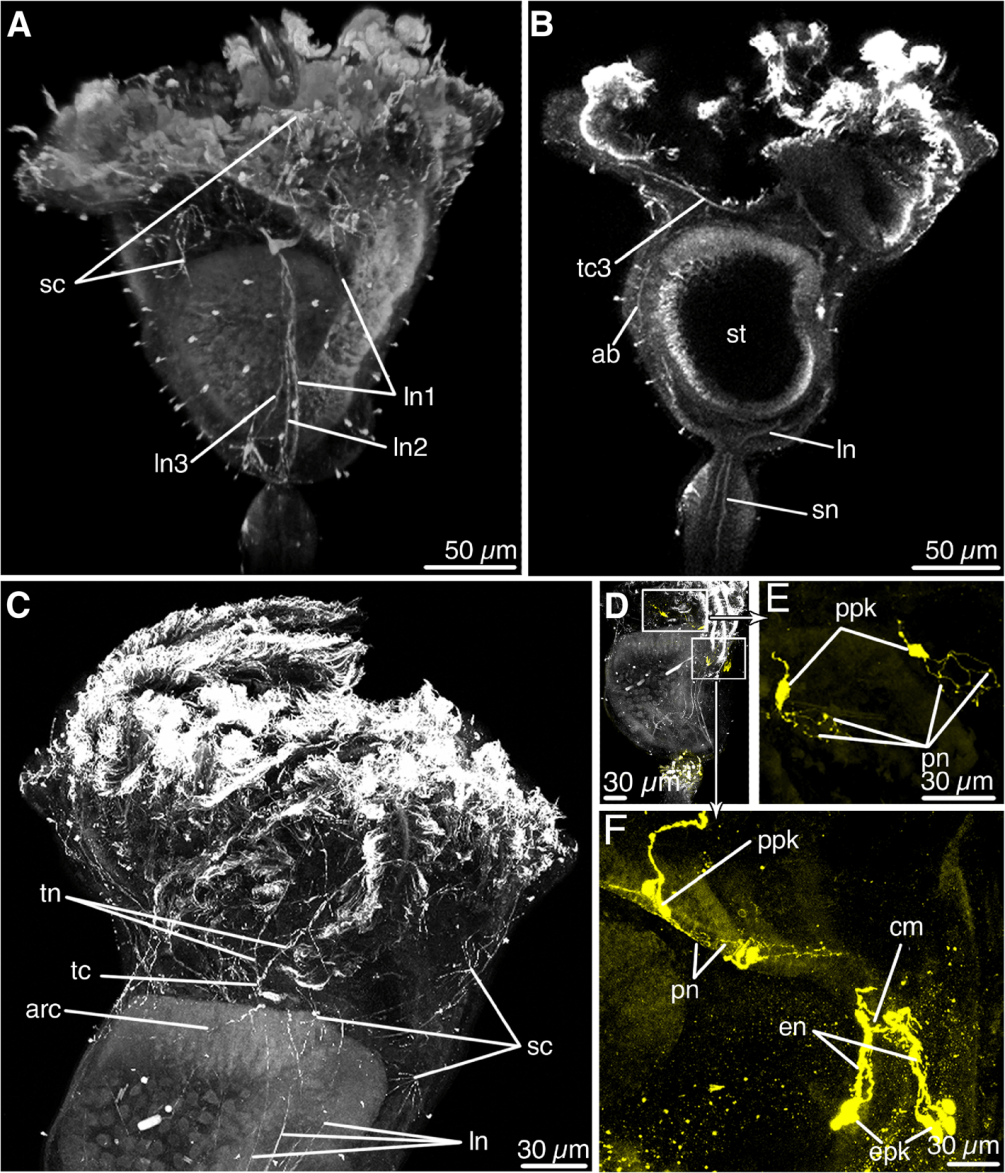
Acetylated α-tubulin-like immunoreactive elements (**a**-**d**) and serotonin-like immunoreactive elements (**d**-**f**) in the nervous system of *Barentsia discreta* according to cytochemistry and laser confocal scanning microscopy. **a** Fronto-lateral view of the calyx and the apical part of the stalk, (**b**) Parasagittal optical section of the calyx showing the aboral nerves (ab) and stalk nerves (sn), (**c**) Lateral view of the upper part of the calyx showing the lateral nerves (ln), arcuate nerves (arc), tentacle nerves (tn), and starlike cells of the calyx (sc), (**d**) Lateral view on the calyx showing the distribution of serotonin-like immunoreactive elements, (**e**) Part of the calyx showing esophageal perikarya (epk) and their neurites (en) with a commissure in between; and perikarya (ppk) and longitudinal peripheral nerves (pn) of the calyx, (**f**) Complex system of neurites (pn) of the large perikarya (ppk) of the calyx. Abbreviations: ab, aboral nerves; arc, arcuate nerves; cm, commissure; en, esophageal nerves; epk, perikarya of esophageal nerves; ln, lateral nerve; ln1, lateral nerve 1; ln2, lateral nerve 2; ln3, lateral nerve 3; pn, peripheral calyx nerves; ppk, perikarya of peripheral nerves; sc, star-like cells; sn, stalk nerve; st, stomach; tc, tentacle cord; tc3, tentacle cord 3; tn, tentacle nerve

**Fig. 4:**
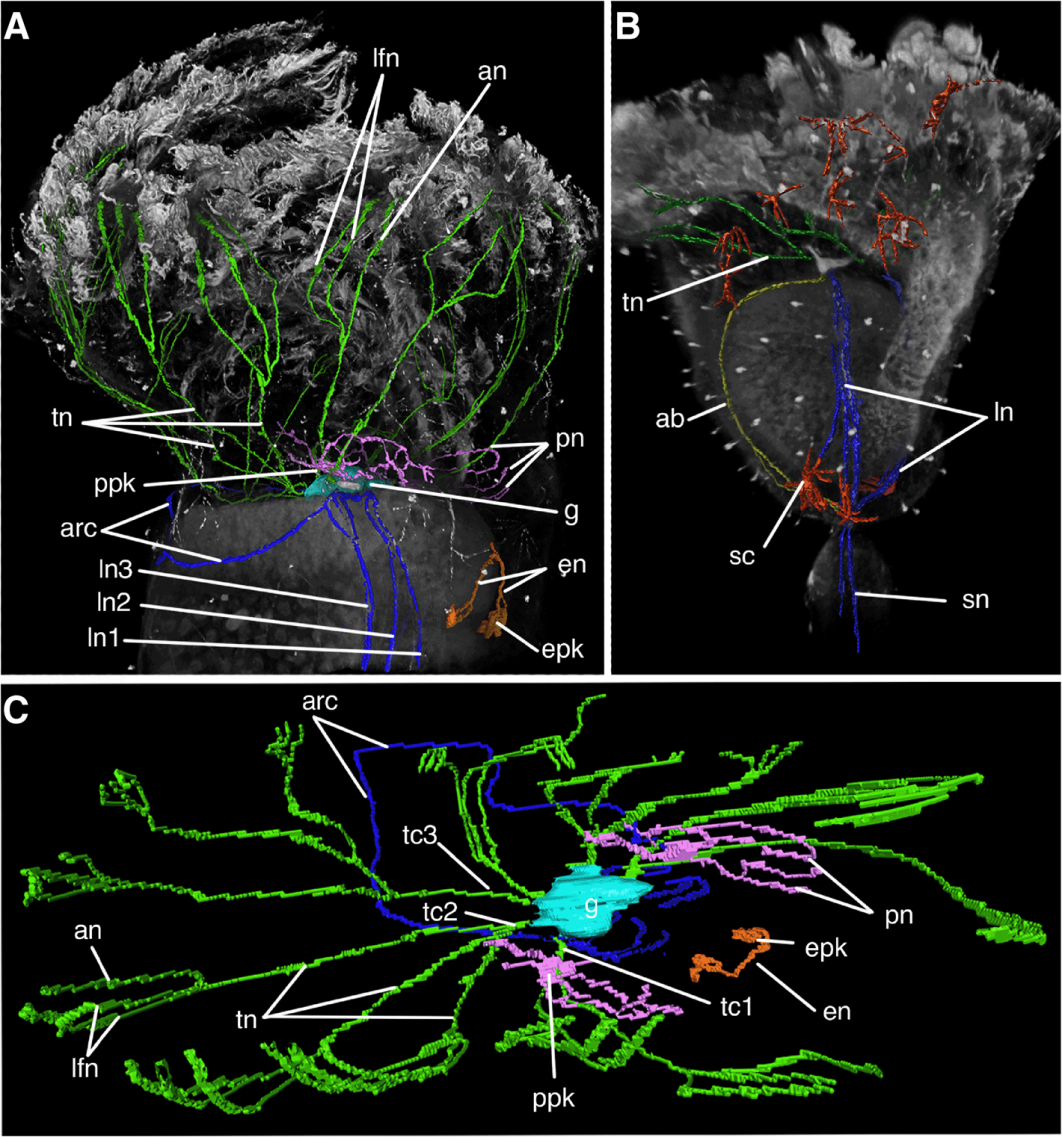
Three-dimensional reconstructions of the nervous system of *Barentsia discreta* after mono- and double staining for 5-HT (serotonin) and acetylated α-tubulin. **a** 3D-reconstruction combined with a volume-rendering of the fronto-lateral view of the upper portion of the calyx, (**b**) 3D-reconstruction combined with a volume-rendering of the fronto-lateral view of the calyx and a portion of the stalk, (**c**) 3D-reconstruction; top view showing innervations of tentacles and serotonergic nerve cells. Abbreviations: ab, aboral nerves; an, abfrontal tentacle nerve; arc, arcuate nerves; en, esophageal nerves; epk, perikarya of esophageal nerves; g, ganglion; lfn, latero-frontal tentacle nerve; ln, lateral nerve; ln1, lateral nerve 1; ln2, lateral nerve 2; ln3, lateral nerve 3; pn, peripheral calyx nerves; ppk, perikarya of peripheral nerves; sc, star-like cells; sn, stalk nerve; tc, tentacle cord; tc1, tentacle cord 1; tc2, tentacle cord 2; tc3, tentacle cord 3; tn, tentacle nerve

Immunostaining with acetylated α-tubulin revealed the presence of several main nerves projecting from the ganglion. Three pairs of *lateral nerves* originate from the sides of the ganglion zone and extend to the base of the calyx (Figs. 3a, c; 4a, b). Lateral nerves pass along the lateral walls of the stomach closer to the esophagus than to the intestine. *Lateral nerve 1* and *lateral nerve 2* pass parallel and close to each other and extend into the area between the calyx and stalk. *Lateral nerve 3* first runs parallel to *lateral nerves 1* and *2,* but at the level of the middle of the stomach, *lateral nerve 3* bends in the abfrontal direction and does not reach the base of the calyx (Figs. 3a; 4b). Paired *lateral nerves 1* merge at the base of the calyx. Where the paired *lateral nerves 1* merge, a new pair of nerves originates, *stalk nerves* that pass through the star-cell complex into the muscular bulb (Figs. 3b; 4b). Two pairs of *aboral nerves* project from the ganglion; they initially extend parallel to the ventral wall of the stomach and then turn at an angle of about 90° and pass on each side of the calyx to its base (Figs. 3b, 4b). In some individuals, a pair of *arcuate nerves* extends toward the aboral side of the calyx from the ganglion zone and merges on the back side of the calyx, forming a semicircular nerve structure (Figs. 3c; 4a, c). Many cells with numerous processes that are stained with acetylated α-tubulin are scattered over the entire surface of the calyx; these are *starlike cells* (Figs. 3a, c, 4b).

The nervous system of *B. discreta* contains a few serotonin-like immunoreactive nerve elements. Two clusters of serotonin-like immunoreactive perikarya are located at the level of lower part of the esophagus. Each cluster includes 3–4 perikarya, from which the processes extend toward the buccal funnel, forming short *esophageal nerves* (Figs. 3d, 4a, c). In the upper part of the esophagus, a comissure is located between two bundles of esophageal nerves (Fig. 3d). The upper part of the calyx contains two large serotonin-like immunoreactive perikarya, which are located on each side of the ganglion but without apparent connection to it (Figs. 3d, e, 4a, c). Each perikaryon forms a net of projections. The long projections of these perikarya extend towards the buccal funnel, whereas the short projections extend towards the intestine. Thus, several pairs of *peripheral calyx nerves* are formed (Figs. 3d, e, 4a, c).

**Fig. 5:**
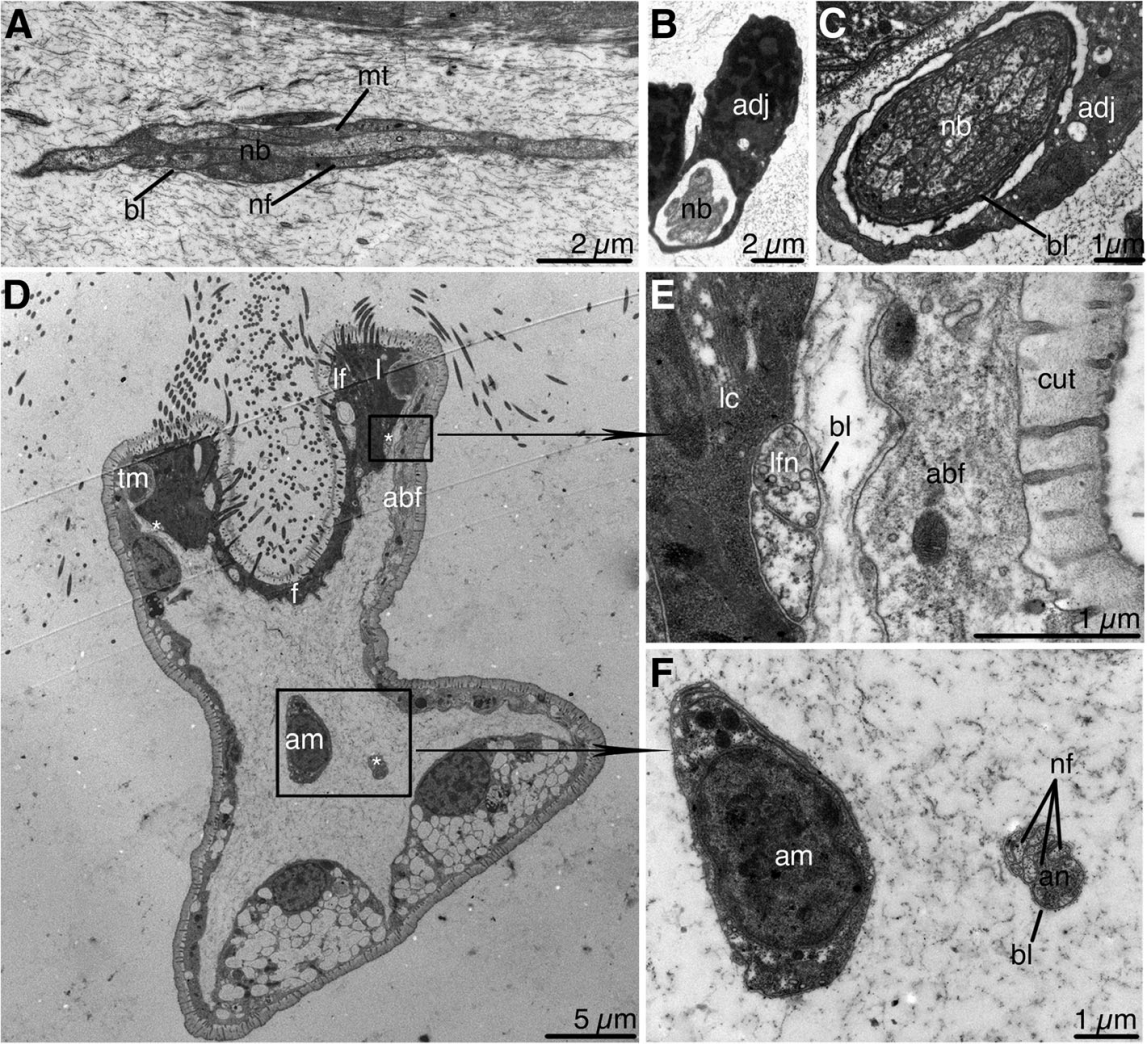
Nerve elements of the calyx and tentacles in *Barentsia discreta*. **a** Longitudinal section through the nerve bundle of the calyx, (**b**), (**c**) Transverse sections through nerve bundles of the calyx, which are surrounded by the processes of the adjacent cell of the body cavity, (**d**) Transverse section of a tentacle (TEM), (**e**) Latero-frontal tentacle nerve (lfn) near the lateral cell of the frontal surface of a tentacle, (**f**) Abfrontal nerve (an) in the cavity of a tentacle. Abbreviations: abf, abfrontal cell of tentacle; adj, adjoining cell of a body cavity; am, amoeboid cell of body cavity; an, abfrontal tentacle nerve; bl, basal lamina; cut, cuticle; f, frontal cell of tentacle; l, lateral cell of tentacle; lf, latero-frontal cell of tentacle, lfn, latero-frontal tentacle nerve; mit, mitochondria; mt, microtubule; nb, neurite bundle; nf, nerve fiber; tm, tentacle muscle;. Asterisks indicate nerve elements in the tentacle

Neurite bundles of the calyx include from 4 to 5 to 50–60 nerve fibers, and the diameter of the bundles ranges from 0.5 to 4.0 μm (Fig. 5a, b, c). Each neurite bundle is surrounded by a thin layer of basal lamina. Some nerves are accompanied by adjoining cells, whose projections form an envelope around the neurite bundle (Fig. 5b, c). The diameter of a separate nerve process in the bundle ranges from 0.15 to 0.5 μm. The cytoplasm of neurites is transparent and contains prominent longitudinally extending microtubules, individual vacuoles, and groups of synaptic vesicles (Fig. 5a).

### Innervation of tentacles

Immunostaining with acetylated α-tubulin revealed three pairs of *tentacular cords* that project from the zone of the ganglion and extend to the tentacle bases (Figs. 3c, 4c). Each *tentacular cord 1* splits into two *tentacle nerves* that innervate the tentacles of the oral side of the calyx. *Tentacular cords 2* and *3* split into three *tentacle nerves* (Figs. 3c, 4a, c). In each tentacle, the nerve is divided into three tentacular neurite bundles: one pair of *latero-frontal tentacle nerves* and one *abfrontal tentacle nerve* (Figs. 4a, c). The *latero-frontal tentacle nerves* are very thin and consist of one or two processes adjacent to the lateral cells of the tentacle (Fig. 5d, e). The *abfrontal tentacle nerve* is located in the cavity of the tentacle near the abfrontal side (Fig. 5d, f). It includes fewer than 8 thin nerve processes, whose cytoplasm contains microtubules, individual synaptic vesicles, and rare mitochondria. Perikarya of nerve cells are not found in the tentacles.

### Sensory organs

**Fig. 6:**
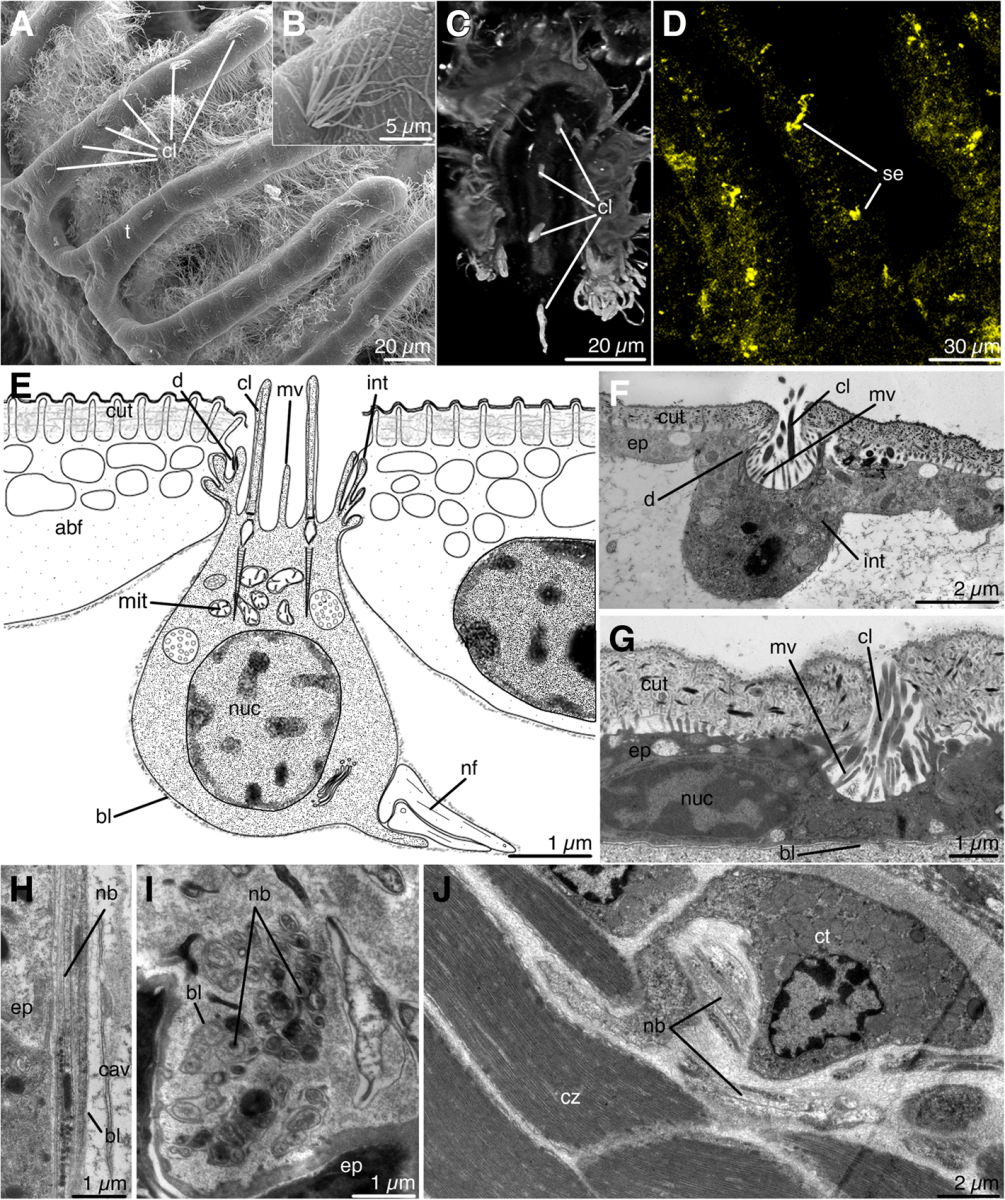
Sensory organs in *Barentsia discreta* and ultrastructure of nerve elements of the stalk in *Barentsia discreta*. **a** Abfrontal surface of a tentacle with bundles of cilia of sensory organs (SEM), (**b**) Cilia of the sensory organ of the tentacle (SEM), (**c**) Abfrontal surface of a tentacle tip showing cilia (cl) of unicellular sensory organs, (CLSM), (**d**) Serotonin-lir elements, whose location corresponds to the location of tentacle sensory organs, (CLSM), (**e**) Schematic structure of epidermal sensory cell, (**f**) Epidermal sensory cell at the base of tentacles, (**g**) Epidermal sensory cell of the apical part of the calyx, near the rectum, (**h**) Longitudinal section through the neurite bundle of the peduncle near the epidermis (ep), (**i**) Transverse section of the neurite bundle in the peduncle, (**j**) Oblique section through the neurite bundle of the muscular base near the cytons of muscle cells (ct). Abbreviations: abfabfrontal cell of tentacle; bl, basal lamina; cav, body cavity; cl, cilia of sensory cell; ct, cyton of muscle cell; cut, cuticle; cz, contractile zone of muscle cell; d, desmosome; ep, epidermal cell; int, interdigitations; mit, mitochondria; mv, microvilli; nb, neurite bundle; nf, nerve fiber; nuc, nucleus; scl, sensory cilia; se, serotonin-lir elements of the tentacle; t, tentacle

Sensory cells occur along the aboral side of tentacles and also at the upper part of the calyx (Fig. 6a, b). On the aboral side of the tentacles, sensory cells are arranged in a row of 4–6 cells, with about 15–30 μm between adjacent cells (Fig. 3d). Each tentacle contains several bundles of cilia that stain with acetylated α-tubulin and that apparently belong to the sensory cells of tentacles (Fig. 6c). Separate serotonin-lir elements are located along the abfrontal surface of the tentacles, whose location corresponds to the location of the tentacle sensory organs (Fig. 6d).

Each sensory organ of the tentacles and calyx is formed by one sensory cell, which is embedded in the epidermis. In most cases, the sensory cell is cone-shaped and protrudes far into the body cavity from the epidermis (Fig. 6e, f); when internal organs come close to the epidermis, however, the sensory cells do not protrude into the body cavity (Fig. 6g). The apical surface of the sensory cells is concave, is not covered by the cuticle, and bears 10–12 cilia and microvilli. A nucleus is located in the basal part of the sensory cell. The cytoplasm of the sensory cell is dense, with a large number of mitochondria, a developed endoplasmic reticulum and Golgi complex, and multivesicular bodies with a diameter of 0.3–0.5 μm. The sensory cells are connected to adjacent epidermal cells by desmosomes and interdigitations. An outgrowth extends from the base of the sensory cell and connects to a nerve bundle (Fig. 6e).

### Nervous system of the stalk

Immunostaining with acetylated α-tubulin revealed the presence of paired *stalk nerves*. These nerves originate at the base of the calyx, pass through the star-cell complex, and continue into the stalk (Figs. 3b, 4b).

In the peduncle, nerve fibers pass into the body cavity, adjoined to the epidermis (Figs. 6h, i). In the muscular base, nerve fibers abut on the muscle cells (Fig. 6j). Each neurite bundle includes 7–10 nerve fibers, which are surrounded by the common basal lamina. Numerous microtubules, synaptic vesicles of different sizes and contents, and mitochondria are located in the transparent cytoplasm of the nerve fibers.

Nerve elements were not detected in the stolon.

## Discussion

### Comparison of the *B. discreta* nervous system with that of other entoprocts

The nervous systems of adult entoprocts have been previously studied in different species by different methods. For example, investigations of living specimens and mounted, picrocarmine-silver-nitrate prepared specimens were used to study the solitary *Loxosoma crassicauda* [19], histological methods were used to study the colonial *Pedicellina cernua* [20], TEM was used to study the solitary *Loxosomella elegans* [21, 23], and immunocytochemistry was used to study the solitary *Loxosomella vivipara* and *L. parguerensis* [23]. Our data, which were obtained by the combined use of histology, TEM, immunocytochemistry, and CLSM, complement and expand previous knowledge, and allow us to derive a general scheme of the organization of the nervous system of adult entoprocts, which we can then compare with the nervous systems of other taxa.

The nervous system of adult entoprocts includes the central ganglion and several paired nerves projecting from the ganglion and extending to the internal organs [26]. The ganglion is a bilaterally symmetric organ consisting of paired lateral lobes that are connected by a transverse commissure. The degree of morphological integration of the paired lobes of the ganglion varies within the Entoprocta. In solitary species, the paired components are widely separated and are referred to as two ganglia connected by a commissure [21, 23]. In colonial forms, including *B. discreta*, the paired components are close together, forming a bilobate or oval ganglion [13, 27]. The greater integration of the nerve ganglion in colonial forms can be regarded as an evolutionarily derived state. Notably, that the colonial species *Loxosomatoides sirindhornae*, which have been regarded as an early offshoot within stolonate entoprocts [28], has a pair of ganglia connected by a commissure [29].

The ganglion of Entoprocta contains peripheral perikarya and a central neuropil [19, 21]. In *B. discreta*, in addition to typical peripheral perikarya, the ganglion contains a second kind of cell, whose function is unclear. Because projections of these cells partly cover the perikarya and separate them from the extracellular matrix, these cells may function as a sort of glial cells that protect and nurse the nerve cells. The presence of accessory cells in the ganglia was noted in other taxa of invertebrate animals, including turbellarians and annelids [30].

The ganglion of *B. discreta* does not exhibit serotonin-like immunoreactivity. The absence of serotonin-like immunoreactivity in the nervous system is not unique to *B. discreta*. For example, the central elements of the nervous system do not exhibit serotonin-like immunoreactivity in perikarya or in neurites in some bryozoans [31, 32]. On the other hand, the absence of the fluorescent signal in the ganglion of *B. discreta* may be the result of methodological limitations, because in loxosomatid species a single serotonergic nerve was described in the ganglion [23].

As shown in previous studies [19, 20, 23] and in the current report, the tentacles of Entoprocta are innervated by several pairs of large nerve cords that extend from the ganglion and then split into tentacle nerves. Small ganglia at the base of tentacles have been described in some species [19]. In *Loxosomella parguerensis*, Fuchs and coauthors [23] described a few large serotonergic perikarya that occur adjacent to the tentacle nerves near the base of some tentacles. However, such perikarya have not been reported in other solitary species or in *B. discreta* in the current study.

Harmer [19] described one nerve in each tentacle of *Loxosoma*, whereas Cori [33] described paired nerves in each tentacle of *Pedicellina*. Nielsen and Rostgaard [24] described a pair of nerves in close contact with the basal part of the lateral cells of the tentacle of *Loxosomella elegans*, and also described cells with many vesicles near the abfrontal sensory organs, which could be considered as nerve cells. Based on immunocytochemical data, Fuchs and coauthors [23] indicated that each tentacle of solitary *Loxosomella* species has three nerves: one RF-amide and a pair of closely spaced, thin, serotonergic fibers. Our data on *B. discreta* confirm the presence of three nerves in each tentacle: two basiephitelial latero-frontal nerves, which are associated with the lateral cells of the frontal surface of the tentacle, and an unpaired subepithelial abfrontal nerve, which runs closer to the abfrontal surface and is apparently associated with the sensory cells of the aboral side of the tentacle. The atrial nerve ring, which is described in solitary entoprocts [23], was not detected in *B. discreta* in the current study. In *B. discreta*, a pair of arcuate nerves extends from the ganglion and merges at the posterior end of the calyx forming a semicircular structure. These nerves have not been found in solitary species.

The innervation of the calyx is mostly similar in *B. discreta* and solitary species, but there are some differences. Although all studied species have lateral and aboral paired nerves, the aboral nerves extend into the stalk in solitary species but terminate at the base of the calyx in *B. discreta*. Only one pair of thin lateral nerves was described in loxosomatids [23], while three pairs of prominent lateral nerves were found in *B. discreta*. Oral nerves, which extend from the ganglion in an oral direction and which then form a loop and continue on the frontal side of the calyx into the stalk, have been described only in solitary species [23]. In *B. discreta*, oral nerves have not been found, but the *lateral nerves 1* of *B. discreta*, which pass close to the esophagus and continue to the stalk, could be homologous with the oral nerves of loxosomatids.

The serotonergic nervous system of *B. discreta* consists of paired clusters of serotonin-like immunoreactive perikarya from which esophageal nerves arise, as well as the perikarya in the upper part of the calyx with their nerve nets forming peripheral nerves. These data only partially corroborate the previous results obtained for solitary species [23]. Loxosomatids have a pair of oral nerves with large perikarya, which are located on the lateral sides of the esophagus. Accordingly to their location, these perikarya are comparable with paired esophageal perikarya of *B. discreta*, but in the latter species, neurites of esophageal perikarya form complex net, which does not connect the cerebral ganglion. The peripheral perikarya and their neurites that are found in the upper portion of the calyx of *B. discreta* cannot be homologized with any serotonin-like immunoreactive nerve elements of solitary species. However, in both *Loxosomella* species [23] several RF-amidergic perikarya with several neurites were found in the upper portion of the calyx. Serotonin-containing elements in the ganglion have been found in solitary species [23], but were not found in *B. discreta* in the current study.

The innervation of the stalk of colonial Entoprocta has been poorly studied. Early authors [13, 22] either did not mention the nerve of the stalk of colonial forms or described a diffuse nerve network [34]. We detected one pair of nerves passing in the peduncle and in the muscular base of the colonial species *B. discreta*, and we demonstrated that the nerves of the stalk are connected with the central ganglion of the calyx. We did not detect a nerve network in the *B. discreta* stalk.

**Fig. 7:**
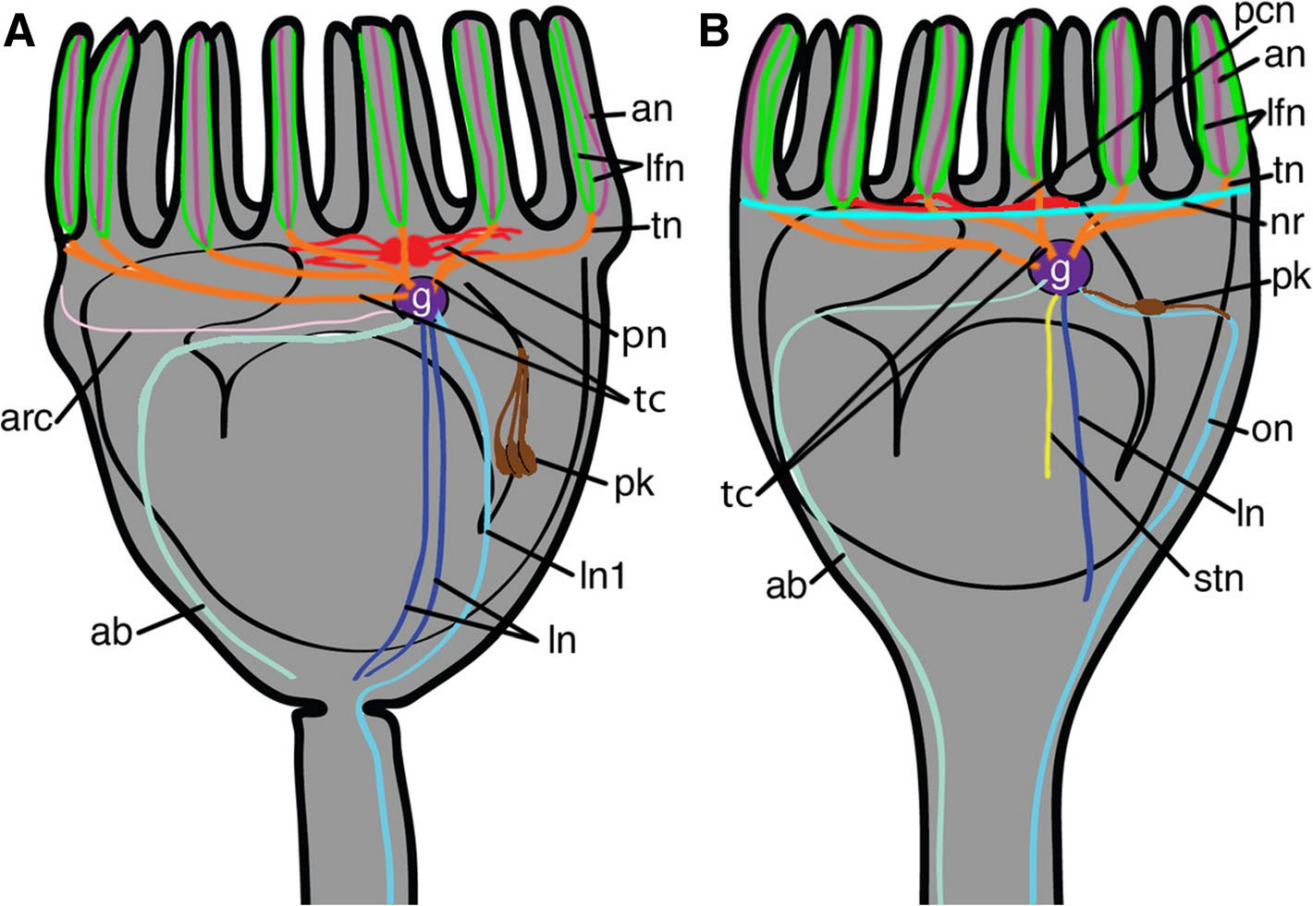
Scheme of the organization of the nervous system of (**a**) colonial and (**b**) solitary Entoprocta. Color legend: orange, tentacle cords and nerves; green, laterofrontal nerve; pink, abfrontal nerve; purple, ganglion; light-green, atrial nerve ring of solitary species; light blue, paired oral nerves of solitary species and the first pair of lateral nerves of colial species; dark blue, paired lateral nerves; mint, paired aboral nerves; lightpink, arcuate nerves of colonial species; yellow, stomach nerve of solitary species; red, peripheral calyx nerves; brown, serotonin-like immunoreactive perikarya connected with esophagus. Abbreviations: an, abfrontal tentacle nerve; arc, arcuate nerve of colonial species; g, ganglion; lfn, latero-frontal tentacle nerve; ln, lateral nerve; ln1, first lateral nerve of colonial species; nr, atrial nerve ring of solitary species; on, oral nerve of solitary species; pn, peripheral calyx nerves; pk, serotonin-like immunoreactive perikarya connected with esophagus; stn, stomach nerve of solitary species; tc, tentacle cord; tn, tentacle nerve

According to the comparative analysis presented above, we developed refined schemes of the organization of the nervous system in different entoprocts (Fig. 7). A paired ganglion is located between the esophagus and the stomach. The ganglion contains two types of cells: perikarya are located along the periphery of the ganglion, whereas neurites occupy the central zone. The ganglion is separated from the body cavity by a thick basal lamina and a layer of adjoining cells. Three pairs of tentacle nerve cords project from the ganglion. Each cord splits into several tentacle nerves. Each tentacle nerve extends to the base of one tentacle, where it divides into three tentacular neurite bundles: a pair of latero-frontal tentacle nerves adjacent to the lateral cells of the tentacle, and an unpaired abfrontal tentacle nerve located near the abfrontal side of the tentacle. Several pairs of neurite bundles project from the ganglion and extend to the base of the calyx and to the stalk (paired oral, lateral, and aboral nerves). Several peripheral calyx perikarya with neurites are located in the upper part of the calyx. Paired serotonergic esophageal perikarya are associated with the esophagus. At least in some species, an atrial nerve ring lies at the base of the tentacles. The atrial nerve ring is not connected to the ganglion.

### Comparison of the nervous system of Entoprocta with that of Bryozoa, Cycliophora, and Mollusca

The relationships between the Entoprocta and Bryozoa have been discussed for several centuries [13, 15, 20, 33, 35–39]. There are many morphological differences in the organization of adults and larvae of entoprocts and bryozoans that have been used by researches to indicate that bryozoans and entoprocts are unrelated taxa [13, 15, 22, 33, 38]. The most prominent difference between adult bryozoans and entoprocts is the location of the anus: it is located inside the tentacles crown in entoprocts but outside in bryozoans. Entoprocta possess protonephridia, while Bryozoa totally lack nephridia. Adult entoprocts are acoelomate, adult bryozoans differentiate true coelomic cavities. Entoprocts have spiral cleavage; bryozoans show a radial cleavage pattern. Another significant difference is the principle of the functioning of the tentacular apparatus: bryozoans are “up-stream filter feeders”, whereas entoprocts are “down-stream filter feeders” [24]. Despite all these differences, some researchers still consider them to be related based on some similarities in their life cycles and especially based on molecular data [11, 12, 40–43]. Nevertheless, morphological criteria supporting such a clade are lacking so far. The organization of the nervous system in the adult and larval stages has traditionally been used for comparative analysis and has played a great role in some phylogenetic conclusions [44–46]. Although the organization of the nervous system exhibits plasticity in many invertebrates, it may help identify homologous structures [47]. The neuroanatomies of Entoprocta and Bryozoa have not been previously compared in detail, although Fuchs and coauthors stated that “immunocytochemical data on postmetamorphic or adult stages of the various lophophorate taxa are scarce and, where available, do not show any obvious positional homologies to entoproct neuronal structures” [23]. Although we can assume that the organization of the nervous system of Entoprocta and Ectoprocta must be different because species from both taxa differ in most other features, it would still be useful to carry out a detailed comparative analysis to confirm or refute this assumption.

**Fig. 8:**
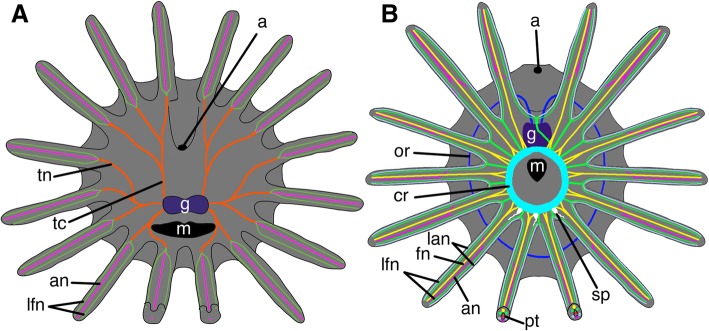
Schemes of the innervations of tentacles in (**a**) entoprocts and (**b**) bryozoans. Color legend: orange, tentacle cords and nerves; green, laterofrontal nerve; pink, abfrontal nerve; purple, ganglion; cyan, circum-oral nerve ring; blue, outer nerve ring; white, oral serotonin-like immunoreactive perikarya; yellow, frontal nerve; grey, lateroabfrontal nerve; red, peritoneal neurites. Abbreviations: a, anal opening; an, abfrontal nerve; cr, circum-oral nerve ring; fn, frontal nerve; g, ganglion; lan, lateroabfrontal nerve; lfn, latero-frontal tentacle nerve; m, mouth; or, outer nerve ring; pt, peritoneal neuritis; sp, oral serotonin-like immunoreactive perikarya; tc, tentacle cord; tn, tentacle nerve

Our data show that the organization of the nervous system is quite different in entoprocts and bryozoans. Both taxa posses a ganglion located next to the anterior gut and several pairs of nerves arise from the ganglion and innervate the body and the tentacle crown. The arrangement of the ganglion differs between these groups. Bryozoans have distinct features of a neuroepithelial organization of the cerebral ganglion. Bryozoan ganglion is essentially a vesicle with distinct cavity delimited by an epithelial layer, which has been shown in both classic and recent works [31, 48–52]. That is why the bryozoan ganglion is traditionally treated as an invagination of the ectodermal epithelium. Entoprocts have no traces of an epithelial organization of the ganglion. The ganglion of entoprocts contains a few cells and is not subdivided into zones, while the ganglion in many bryozoans has three regions [31, 53–55]. The peripheral nervous system of bryozoans is considered to be mostly basiepidermal or interepidermal with a diffuse epidermal nerve plexuses in the body, whereas entoprocts have a subepidermal nervous system without nerve plexuses. Although both bryozoans and entoprocts have sensory cells arranged in rows along the abfrontal side of the tentacles, the structure of these organs differs in the two groups. In bryozoans, sensory cells of tentacles are conical, with a narrow apical surface that is covered with the cuticle [56]. Sensory cells in entoprocts, in contrast, have a concave apical surface and are not covered by the cuticle (Fig. 6e, f, g). There are obvious differences in the innervation of the tentacles of Bryozoa and Entoprocta (Fig. 8). In bryozoans, tentacles are innervated from the circum-oral (or circum-pharyngeal) nerve ring. Characteristically, some nerves in bryozoans do not extend from the nerve ring directly into the tentacle, but instead extend into the intertentacular membrane, where they branch toward the two adjacent tentacles [53, 57–59]. In general, four longitudinal basiepidermal nerves are found in each tentacle of bryozoans: one frontal nerve, one abfrontal nerve, and one pair of laterofrontal nerves. In some bryozoans, six or two nerves have been detected in each tentacle, but never three nerves as is the case for entoprocts [31, 60]. In entoprocts, tentacles are innervated not by the nerve ring but by three pairs of tentacular cords arising from the ganglion. Each tentacle is innervated from one nerve, which branches at the base of the tentacle into three bundles: two basiepidermal latero-frontal tentacle nerves and one subepidermal abfrontal tentacular nerve. There is one detail in the innervation of the tentacles that seems similar in the Bryozoa and Entoprocta. If we trace the innervation of tentacles of Bryozoa, we can see that the tentacles of the oral and anal sides of the lophophore are innervated from different structures. This is particularly noticeable for the Phylactolaemates, in which the anal tentacles are innervated by the lophophore nerve horns, while the oral tentacles are innervated from the circum-pharyngeal nerve ring [57]. In addition, several radial nerves extend to the lateral tentacles directly from the ganglion as seen in the reconstruction of zooid innervation (see [57]: Fig. 1). Gymnolaemata also have traces of this separate innervation, i.e., the anal tentacles are innervated by lophophoral dorso-lateral nerves, while other tentacles are innervated by the nerve ring [31]. The circum-pharyngeal ring may not be closed in some bryozoans [57], and may therefore be represented by only one pair of nerves bending around the mouth. Accordingly, we can conclude that the bryozoan ganglion gives rise to several pairs of nerves that then branch and extend into the tentacles. Entoprocta also have several pairs of nerve cords that project from the ganglion: one pair bends around the oral opening and extends to the oral tentacles; another innervates the lateral tentacles; and the last extends to the anal tentacles. This similarity may correlate with a more or less similar position of mouth and anus in these groups, and does not outweigh the differences.

It would be interesting to compare the nervous system of Entoprocta with that of Cycliophora, which is considered to be a sister group to Entoprocta [1–5]. The organization of the nervous system in the feeding stage of Cycliophora, however, is still unknown in detail. The cycliophoran feeding stage presumably possesses one ganglion located near the esophagus and one ganglion at the base of the buccal funnel with a pair of lateral nerves running to the mouth ring [61]. The ultrastructure of the ganglion has not been described. According to brief TEM descriptions, cycliophoran feeding stages have aggregations of nerve fibers in the buccal funnel, at the base of the mouth ring, and between the upper part of stomach and the anus. Data based on immunocytochemical methods are inconsistent and not useful for comparisons because of scattered and weak signal [61, 62]. Sensory organs have not been described in the cycliophoran feeding stage [61]. Because the information on the organization of the nervous system in cycliophorans is fragmentary, a detailed and useful comparative analysis is not possible at this time.

The close relationship between Entoprocta and Mollusca was first suggested at the end of the twentieth century based on the presence of the sinus of circulatory system in both groups [63, 64]. Later, some common features were discovered in the morphology of creeping larvae of entoprocts and the larvae and adults of “basal mollusks” [16–18]. Although most molecular data do not support the unity of Entoprocta and Mollusca, a recent study by Marlétaz and coauthors [65] indicated that mollusks and entoprocts form a monophyletic clade. This clade, however, did not have very high support and the phylogenetic tree was reconstructed without consideration of cycliophorans, which are usually regarded as close relatives of entoprocts [1–5].

According to morphological studies [16, 17], the unity of the mollusc–entoproct clade is supported by the organization of the nervous system in entoproct creeping larvae and in larvae and adults of “basal mollusks”. Although similarities in the nervous systems of the two groups should be supported by new data from as yet unstudied species, it is interesting to find any similarities in the organization of the nervous systems of mollusks and adult entoprocts. Considering that Entoprocta is regarded as a neotenic group [10, 40, 66], we can assume that at least some features of the larval nervous system are retained in adults, and that there is a possibility of finding homology between adult entoprocts and adult “basal mollusks”. Some larval features, including the digestive system [67], are definitely preserved in adult entoprocts. Unfortunately, almost nothing is known about the transformation of the nervous system during metamorphosis. Research has determined that the larval ganglia are destroyed and that the ganglion in adults is formed de novo [10, 13]. At the same time, some authors [27, 68] described a larval subesophageal ganglion, which is maintained in metamorphosis and which functions as the cerebral ganglion of adult entoprocts. There is no information about the transformations of the main nerves of the larvae during metamorphosis. We could attempt to directly compare the organization of the larval and adult nervous systems, but that kind of comparison seems to be quite speculative without additional study of the transformation of the larval nervous system into the definitive nervous system of the juvenile. Moreover, the data on the neuroanatomy of the entoprocts larvae are limited to only the creeping larva of the solitary *Loxosomella murmanica* [16].

We therefore must conclude that, given the current level of morphological knowledge, it is impossible to determine with certainty whether any elements in the nervous systems of adult entoprocts and adult “basal mollusks” are homologous.

## Conclusions

The organization of the nervous system of colonial and solitary entoproct species has a similar plan, but differs in details of the ultrastructure of the ganglion, the number of neurite bundles in the calyx, and the distribution of serotonin in the nerve elements. In general, the nervous system of entoprocts consists of several pairs of large nerve cords extending from the ganglion and then splitting into tentacle nerves, and several pairs of longitudinal nerves (lateral and aboral nerves) extending from the ganglion and innervating the body. The nervous system is considered to be a rather conservative organ system in many invertebrate phyla, and therefore can be used for phylogenetic reconstructions [47, 69, 70]. As documented in the current study, there are many differences in the neuroanatomy of bryozoans and entoprocts, including the innervation of the tentacular apparatus. The morphology of the tentacular apparatus is also substantially different in bryozoans and entoprocts. Taking together, different morphology and innervation of the tentacular apparatus may indicate an independent origin of the tentacular apparatus, and the absence of a close relationship between Bryozoa and Entoprocta. On the other hand, these differences might have developed from a common state as a result of specificities of life history and life style: entoprocts evolved as a neotenic group [10, 15, 68]; bryozoans evolved as colonial animals. With these differences in life history and life style, the neuroanatomy of entoprocts and bryozoans may have increasingly diverged over time. The comparison of the organization of the nervous system of entoprocts with other taxa that are considered to be possible related to Entoprocta (Cycliophora and Mollusca) is not really possible at this time due to lack of morphological data.

## Methods

### Sampling of animals

Colonies of *Barentsia discreta* (Busk, 1886) were collected from the shells of the bivalve *Modiolus modiolus* (L., 1758) in August of 2009, 2015, and 2016, in the Peter the Great Bay of the Sea of Japan at depths of 3–15 m. All individuals were relaxed in a solution of 7% MgCl_2_ and fixed in a solution of 2.5% glutaraldehyde in 0.1 M sodium phosphate buffer (PBS) for future studies by different methods.

### Light microscopy

For histological studies, nine specimens of *B. discreta* were washed in distilled water, dehydrated in an ethanol series of increasing concentration, in 96% ethanol mixed with butanol, and in pure butanol, and then embedded in paraplast. Then specimens were cut with microtome Leica RM 2125 (Leica, Germany) (thickness of slice is 4 μm). Series of sagittal and transversal histological sections were stained with hematoxylin and then mounted in a Canadian balsam. The sections were photographed with the AxioCam HRm camera, using the Zeiss Axioplan 2 microscope.

### Transmission electron microscopy (TEM)

For TEM, samples were postfixated in a solution of 1% OsO_4_ in PBS. The samples were then dehydrated in an ethanol series of increasing concentration, in 96% ethanol mixed with acetone, and in pure acetone. For TEM, the dehydrated samples were embedded in epoxy resin (EPON). Ultrathin sections were cut on an ultratome (Leica EM UC6) and then stained for 40 min with saturated uranyl acetate and for 7 min with lead citrate. The sections were examined and photographed with a JEOL JEM-1011 and JEM-100B transmission electron microscope.

### Scanning electron microscopy (SEM)

For SEM, the material was fixed and dehydrated as described for TEM. The dehydrated material was transferred into liquid CO_2_ and critical point dried. The dried specimens were sputter-coated with platinum–palladium and examined with a JEOL JSM 6380 scanning electron microscope.

### Immunocytochemistry and confocal laser scanning microscopy (CLSM)

For immunocytochemical staining, eight specimens were fixed in 4% PFA in 0.1 M PBS overnight at 4 °C. They were then washed three times for 15 min in 0.1 M PBS and stored in 0.1 М PBS with 0.03% NaN_3_. For serotonin and acetylated a-tubulin double labelling, the material was first permeabilized in 0.01 M PBS with 0.03% NaN_3_ and 5% Triton X-100 for 2 days at 4 °C. To block unspecific binding sites, specimens were transferred to 0.01 M PBS with 0.03% NaN_3_ and 1% Triton X-100 with 1% bovine serum albumin (BSA) for 1 day at 4 °C. The specimens were incubated in the first antibodies, i.e., in a mixture of anti-serotonin (rabbit polyclonal, 1:1000; Chemicon, Temecula, CA, USA) and anti-acetylated α-tubulin (mouse monoclonal, 1:1600; Sigma, USA) antibodies. Primary antibodies were applied at 1:700 dilution in 0.01 M PBS with 0.03% NaN_3_, 1% Triton X-100, and 1% BSA for 24 h at 4 °C. The specimens were then washed several times in PBS and incubated in a cocktail of secondary antibodies (Goat Anti-Rabbit Alexa IgG Antibodies labeled with Alexa Fluor 488 and Goat Anti-Mouse IgG Antibodies labeled with Alexa Fluor 632, 1:1000 in 0,1 M PBS, and 1% Triton Х100) for 24 h at 4 °C. The material was then washed in 0.1 M PBS and stored in a 1:1 mixture of glycerol and PBS. Optical sections were digitally recorded using a Nikon A1 confocal laser scanning microscope. Images were processed using ImageJ software and Amira version 5.2.2 software (Thermo Fisher Scientific, MA, USA). 3D reconstructions were performed using Amira version 5.2.2 (used tools are Voltex, LabelField,and SurfaceGen).

## Abbreviations

a: anal opening
ab: aboral nerves
abf: abfrontal cell of tentacle
ac: anal cone
adj: adjoining cell of a body cavity
am: amoeboid cell of body cavity
an: abfrontal tentacle nerve
arc: arcuate nerves
b: bud
bf: buccal funnel
bl: basal lamina
c: calyx
cav: body cavity
cl: cilia of sensory cell
cm: commissure
cr: circum-oral nerve ring
ct: cyton of muscle cell
cut: cuticle
cz: contractile zone of muscle cell
d: desmosome
dc: dark cell of ganglion
en: esophageal nerves
ep: epidermal cell
epk: perikarya of esophageal nerves
es: esophagus
f: frontal cell of tentacle
fn: frontal nerve
g: ganglion
gd: gonoduct
in: intestine
int: interdigitations
l: lateral cell of tentacle
lan: lateroabfrontal nerve
lf: latero-frontal cell of tentacle
lfn: latero-frontal tentacle nerve
ll: lower lip
ln: lateral nerve
ln1: lateral nerve 1
ln2: lateral nerve 2
ln3: lateral nerve 3
m: mouth
mb: muscular base
mbl: muscular bulbous
mit: mitochondria
mt: microtubule
mv: microvilli
n: nerve cell
nb: neurite bundle
nf: nerve fiber
nph: nephropore
nr: atrial nerve ring
nrp: neuropil
nuc: nucleus
on: oral nerve
or: outer nerve ring
p: peduncle
pk: serotonin-like immunoreactive perikarya connected with esophagus
pn: peripheral calyx nerves
ppk: perikarya of peripheral nerves
pt: peritoneal neuritis
rec: rectum
s: stolon
sc: star-like cells
scl: sensory cilia
se: serotonin-lir elements of the tentacle
sn: stalk nerve
sp: oral serotonin-like immunoreactive perikarya
st: stomach
stn: stomach nerve; stc, star-cell complex
t: tentacle
tc: tentacle cord
tc1: tentacle cord 1
tc2: tentacle cord 2
tc3: tentacle cord 3
tm: tentacle muscle
tn: tentacle nerve
vg: vestibular groove
